# Anti‐obesity effects exerted by *Dioscorea opposita* Thunb. polysaccharides in diet‐induced obese mice

**DOI:** 10.1002/fsn3.3588

**Published:** 2023-08-06

**Authors:** Sheng‐Nan Li, Dian‐Long Zhang, Zhen‐Hui Wang, Wen‐Ting Song, Wen‐Bo Chen, Ge‐Li Hu, Lu‐Ying Han, Jing‐Chun Zhou

**Affiliations:** ^1^ School of Medicine Henan Polytechnic University Jiaozuo China; ^2^ Beijing Bencaoyuan Pharmaceutical Co., Ltd Beijing China

**Keywords:** *Dioscorea opposita* Thunb., gut microbiota, obesity, polysaccharides

## Abstract

Obesity is characterized by chronic inflammation, insulin resistance, and gut microbiota dysbiosis. *Dioscorea opposita* Thunb. is a traditional food and medicine homolog from China. In the present study, polysaccharides isolated from a water extract of *Dioscorea opposita* Thunb. (DOTPs) were prepared. We showed that DOTPs reduced body weight, accumulation of fat tissues, insulin resistance, and inflammation in high‐fat diet (HFD)‐fed mice. Further experiments showed that DOTPs could regulate the composition of the gut microbiota in HFD mice. DOTPs supplementation in HFD‐fed mice resulted in the reduction of the *Firmicutes*‐to‐*Bacteroidetes* ratio. We further demonstrated that DOTPs supplementation enhanced bacterial levels of *Akkermansia* and reduced levels of *Ruminiclostridium_9*. A significant reduction of glycolysis metabolism related to obesity and gut microbiota dysbiosis was also observed upon administration of DOTPs. Our results suggest that DOTPs can produce significant anti‐obesity effects, by inhibiting systematic inflammation and ameliorating gut microbiota dysbiosis in diet‐induced obese mice.

## Graphical abstract

For enhancing the economic value of an edible Dioscorea opposita Thunb., the polysaccharides (DOTPs) were isolated. We found that DOTPs produced significant anti‐obesity effects in diet‐induced obese mice. Thus, DOTPs may be a potential prebiotic agent for the treatment of obesity.

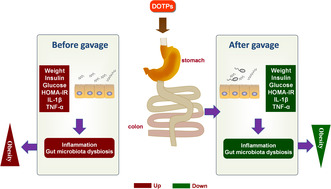

## 1 INTRODUCTION

Obesity, a disease condition, is associated with the increased incidence of specific diseases, including type 2 diabetes mellitus, fatty liver disease, inflammation, fat metabolic disorders, cardiovascular disease, and some cancer types (Pillon et al., 2021). COVID‐19 patients with obesity or type 2 diabetes exhibit a higher propensity for disease exacerbation (Barron et al., 2020). Obesity is characterized by excessive body weight gain, accumulation of fat tissue, adipocyte hypertrophy, gut microbiota dysbiosis, chronic inflammation, insulin resistance, and metabolic endotoxemia (Cani et al., 2008; Morris et al., 2013; Turnbaugh et al., 2006). Adipose tissue, the main executor of chronic inflammation in obesity, can secrete tumor necrosis factor (TNF‐α) and interleukin‐6 (IL‐6), which play a negative role in regulating the insulin signaling pathway, ultimately resulting in insulin resistance. Since obesity can promote insulin resistance, it is also a major factor driving the global prevalence of diabetes (Siwicki et al., 2016).

The gut microbiota comprises trillions of bacteria, which can be considered a giant metabolic secretory organ in organisms, as well as an important regulator of host metabolism (Bäckhed et al., 2004; Canfora et al., 2019; Dethlefsen et al., 2007). The abnormal composition or function of the gut microbiota can contribute to host metabolic disorders, including alterations in the metabolism of the adipose tissue. In healthy individuals, the composition of the gut microbiota is diverse, while in obese individuals, its diversity is decreased (Bäckhed et al., 2004; Liu et al., 2017; Turnbaugh et al., 2009). The effect of an altered dietary intake on the gut microbiota is closely related to the occurrence of obesity (Koeth et al., 2013; Mozaffarian et al., 2011). Some studies have shown that the modulation of the gut microbiota using prebiotics could reduce chronic inflammation and obesity (Depommier et al., 2019; Wang et al., 2015; Yu et al., 2020).

Specific types of Traditional Chinese Medicine (TCM) used as foods have been utilized for thousands of years in Asia, to prevent and treat various diseases. Their unique advantages and efficacy have increasingly gained recognition and appreciation from the international medical community. The “multi‐component for multi‐target” action mode of TCM has gained increased acceptance. The search for TCM or its components, as a potential treatment for obesity, has become a new research focus. Different chemical components of TCM, such as alkaloids, phenols, and polysaccharides, could inhibit obesity by regulating the gut microbiota (Li et al., 2016; Zhang et al., 2012). The fungus *Ophiocordyceps sinensis* and its anamorph *Hirsutella sinensis* have a long history of utilization in TCM. In HFD‐fed mice, the neomycin‐sensitive bacteria *Parabacteroides goldsteinii* were essential for the anti‐obesity effects of the polysaccharides extracted from the *Hirsutella sinensis* mycelium (Wu et al., 2019). *Ganoderma lucidum* is a frequently used herb in TCM. High‐molecular‐weight polysaccharides extracted from *Ganoderma lucidum* could reduce metabolic endotoxemia, obesity, inflammation, and insulin resistance in mice, by modulating the composition of the gut microbiota (Chang et al., 2015).

*Dioscorea opposita* Thunb. which is produced in Jiaozuo City, Henan Province, is a traditional food and medicine homolog in China. *Dioscorea opposita* Thunb. is rich in allantoin, flavonols, proteins, saponins, and polysaccharides. Polysaccharides are considered one of the main bioactive compounds of *Dioscorea opposita* Thunb., demonstrating anti‐tumor, anti‐oxidation, anti‐aging, immunomodulatory, and hypoglycemic effects (Fan et al., 2015; Niu et al., 2017; Wang, Shi, & Wang, 2018; Wu et al., 2018; Zhi et al., 2019). The polysaccharides DOTP‐B, DOTP80, and glucomannan were isolated from a water extract of *Dioscorea opposita* Thunb. (DOT). The monosaccharides found in these polysaccharides include glucose, mannose, galactose, xylose, arabinose, and rhamnose (Fan et al., 2015; Ma et al., 2017; Zhi et al., 2019). In the present study, we showed that DOT and polysaccharides isolated from the extract (DOTPs) reduced the body weight and the accumulation of fat tissues in HFD‐fed mice. Further experiments demonstrated that DOTPs could regulate the composition of the gut microbiota in HFD mice. Therefore, our findings suggest that DOTPs could be a potential prebiotic agent for the treatment of obesity.

## 2 MATERIALS AND METHODS

### 2.1 Animals

Forty 4‐week‐old male C57BL/6J mice were purchased from the Experimental Animal Center of the Zhengzhou University (Zhengzhou, Henan Province, China). Mice were housed in a controlled environment, under a 12‐h light–dark cycle, with 22 ± 2°C temperature and 50 ± 5% relative humidity. After one week of acclimatization, mice were randomly distributed into five groups (with two or three individuals per cage). Animals were fed for 12 weeks with either a standard chow diet (Chow, 13.6 kcal% fat; LabDiet; USA) or a high‐fat diet (HFD, 60 kcal% fat; Research Diets D12492; USA). Mice were daily supplemented with either 100 μL sterile saline solution, 60 mg/kg DOT, 60 mg/kg DOTPs‐1, or 30 mg/kg DOTPs‐2, by intragastric gavage. Food consumption and body weight were recorded every seven days. This study was approved by the Medical Ethics Committee of the Henan Polytechnic University (approval code: HPU‐MEC‐2020‐04). All procedures used in this study complied with the Guide for the Care and Use of Laboratory Animals of the Henan Polytechnic University.

### 2.2 Preparation of *Dioscorea opposita* Thunb. water extract and polysaccharides

*Dioscorea opposita* Thunb. as a TCM is distributed in the area of Jiaozuo City, Henan Province, China. Fresh *Dioscorea opposita* Thunb. was peeled, diced into small pieces, and placed under a constant temperature oven at 60°C, until it was completely dried. Then they were comminuted into powder. The powder was mixed with pure water (at a w/w ratio of 1:30), followed by intermittent agitation at 60°C for 2 h. The supernatant was obtained after centrifugation at 5000 rpm for 15 min. The sediments were blended with pure water (at a w/w ratio of 1:30), followed by a second intermittent agitation step at 60°C for 2 h. The supernatant was obtained after centrifugation. The twice‐combined supernatant was vacuumed to dry at 70°C to obtain DOT. Then, it was sub‐packed and stored in a refrigerator (−20°C). As described above, the DOT supernatant was treated with pure alcohol (at a v/v ratio of 1:4) for 12 h. The solution was centrifuged at 4000 rpm for 15 min at 4°C. The sediments were collected and subsequently washed with pure alcohol, followed by centrifugation. The precipitate pellet, containing crude polysaccharides, was thoroughly dissolved in water. The crude polysaccharides (at a v/v ratio of 4:1) were washed with Sevage reagent (a 5:1 mixture of chloroform and n‐butyl alcohol). The protein content was eluted by full oscillation for 30 min, followed by centrifugation at 4000 rpm for 10 min. The obtained upper suspension was a polysaccharide solution. Pure alcohol (at a v/v ratio of 4: 1) was added to the polysaccharide solution, followed by centrifugation at 4000 rpm for 10 min. The sediments were vacuumed to dry at 70°C to obtain the DOTPs, which were subsequently sub‐packed and stored in a refrigerator (−20°C). In summary, 0.3 g DOTPs were obtained from 2.76 g DOT, with a yield of 10.9%.

### 2.3 Hematoxylin–eosin (H&E) staining

For histological analysis, adipose tissues were fixed in 10% neutral buffered formalin for 24 h at room temperature, followed by dehydration and paraffin embedding. Then, tissues were sectioned into thick slices (7 μm). Following dewaxing and rehydration, sections were stained in Harris hematoxylin solution for 5 minutes and subsequently washed in running tap water. Then, sections were stained in eosin solution, followed by washing in water and microscopic imaging.

### 2.4 Cytokine quantifications

The blood collected from the mouse eye socket was allowed to coagulate at room temperature for 30 min. The serum was obtained by centrifugation at 1000 *g* for 15 min. The levels of interleukin‐1β (IL‐1β) protein, TNF‐α protein, and fasting insulin were measured using commercial enzyme‐linked immunosorbent assay (ELISA) kits, whose readouts were read at 450 nm with a microplate reader. Fasting levels of blood glucose were quantified using glucometer strips (ACCU‐CHEK Performa, Roche). The homeostatic model assessment‐insulin resistance (HOMA‐IR) index was calculated using the following equation: fasting glucose (mmol/L) × fasting insulin (μU/mL)/22.5 (Kernan et al., 2016; Matthews et al., 1985).

### 2.5 Gut microbiota analysis

A random selection of five mice in each group was used for the bioinformatics analyses by LC‐Bio Technology Co., Ltd. (Hangzhou, Zhejiang Province, China). The E.Z.N.A.® Stool DNA Kit (Omega Bio‐Tek, USA) was used for microbial DNA extraction. We amplified the bacterial 16S rDNA using a primer set specific for its V3–V4 variable region, with the sequences: 341F (5′‐CCTACGGGNGGCWGCAG‐3′) and 805R (5′‐GACTACHVGGGTATCTAATCC‐3′). Sample sequencing was performed using an Illumina platform (Illumina, USA). The high‐quality clean tags were obtained after chimeric sequence filtering and quality control. After dereplication using DADA2, we obtained a feature table and feature sequences. The microbiota analyses were assessed using QIIME2‐based analysis.

### 2.6 Statistical analysis

The data were analyzed using GraphPad Prism software (GraphPad Software, USA). All results were expressed as the means ± standard deviations (SD). Differences between two groups were assessed using the unpaired two‐tailed Student's *t*‐test. Data sets involving multiple groups were assessed by one‐way analysis of variance followed by Tukey's multiple comparisons test. A value of *p* < .05 was considered statistically significant.

## 3 RESULTS

### 3.1 DOTPs reduced body weight in HFD‐fed mice

**FIGURE 1:**
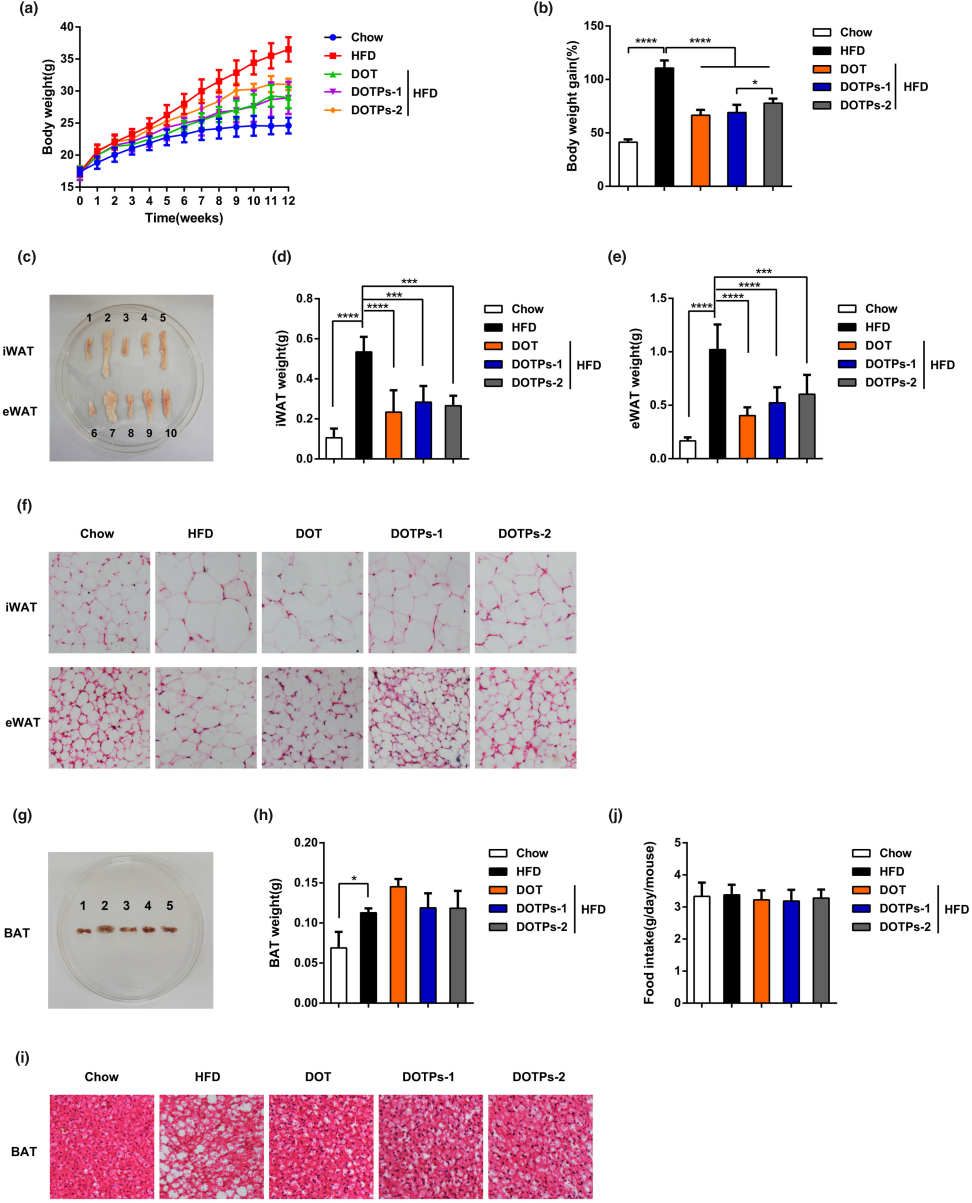
DOTPs reduced body weight and fat accumulation in HFD‐fed mice. Chow‐fed and HFD‐fed mice were treated daily for 12 weeks, with either a saline solution, 60 mg/kg DOT, 60 mg/kg DOTPs‐1, or 30 mg/kg DOTPs‐2, by intragastric gavage (*n* = 8 for each group). (a) The body weight and (b) body weight gain were measured. (c) Representative images of iWAT and eWAT (1 or 6 for Chow; 2 or 7 for HFD; 3 or 8 for DOT; 4 or 9 for DOTPs‐1; 5 or 10 for DOTPs‐2). (d) iWAT weight and (e) eWAT weight were measured. (f) Representative images of H&E staining of iWAT and eWAT. (g) Representative images of BAT (1 for Chow; 2 for HFD; 3 for DOT; 4 for DOTPs‐1; 5 for DOTPs‐2). (h) BAT weight was measured. (i) Representative images of H&E staining of BAT. (j) Food intake. Data are presented as mean ± SD of three independent experiments (*p < .05, **p < .01, ***p < .001, ****p < .0001).

We fed mice with an HFD for 12 weeks and supplemented DOT (or a saline solution as a negative control) via intragastric gavage. In comparison with mice fed a chow diet, mice fed an HFD showed significant increases in body weight, epididymal white adipose tissue (eWAT) mass, inguinal white adipose tissue (iWAT) mass, brown adipose tissue (BAT) mass, and adipocyte size (Figure 1a–i). It was noteworthy that the supplementation with DOT decreased these obesity traits in HFD‐fed mice (Figure 1a–i). Subsequently, we found that the polysaccharides in DOT had anti‐obesity effects. In comparison with HFD, administration of DOTPs reduced the body weight by approximately 20% after 12 weeks (Figure 1a). DOTPs also reduced the masses of eWAT and iWAT (but not of BAT) and decreased adipocyte size in HFD‐fed mice, producing similar effects to those observed for the DOT group (Figure 1c–i). Furthermore, no difference in food intake was observed between the groups (Figure 1j). However, in the DOTPs and DOT groups, food intake tended to slightly decrease, in comparison with the HFD group.

### 3.2 DOTPs reduced insulin resistance in HFD‐fed mice

**FIGURE 2:**
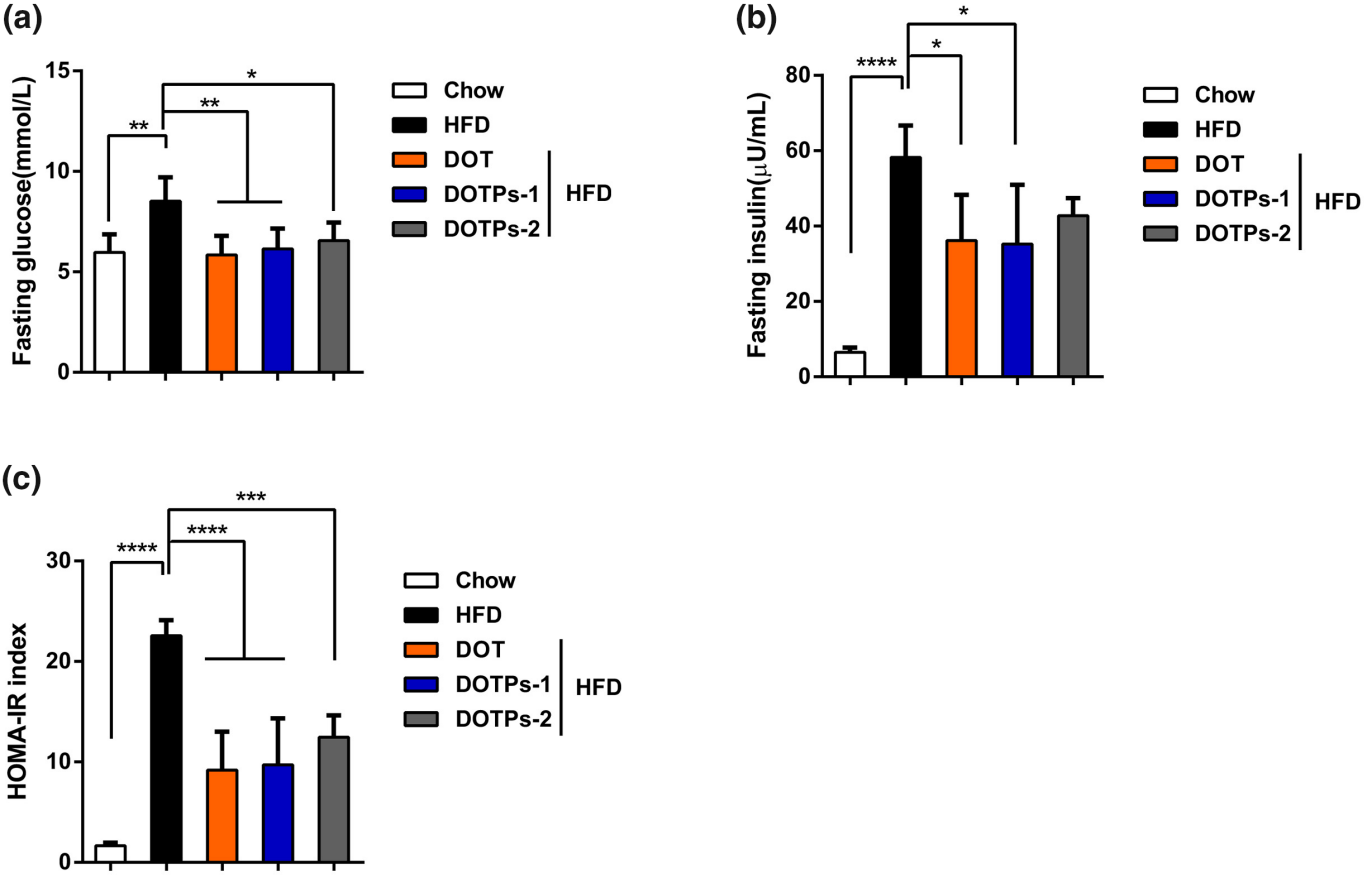
DOTPs restored glucose and insulin resistance in HFD‐fed mice. The fasting blood levels of glucose and insulin were measured after 12 weeks of treatment as described in Figure 1. Insulin resistance was assessed by the HOMA‐IR index. (a) Levels of fasting blood glucose and (b) levels of fasting serum insulin were measured. (c) The HOMA‐IR index was estimated. Data are presented as the mean ± SD of three independent experiments (*p < .05, **p < .01, ***p < .001, ****p < .0001).

To assess insulin resistance, we selected the HOMA‐IR index. In comparison with mice fed a chow diet, mice fed an HFD showed significant increases in fasting blood levels of glucose, insulin, as well as in HOMA‐IR levels (Figure 2a–c). Supplementation with DOT and DOTPs resulted in lower levels of blood glucose, insulin, and HOMA‐IR in HFD‐fed mice (Figure 2a–c). These results indicated that the HFD‐fed mice exhibited insulin resistance, which could be prevented through the administration of DOTPs.

### 3.3 DOTPs reduced inflammation in HFD‐fed mice

**FIGURE 3:**
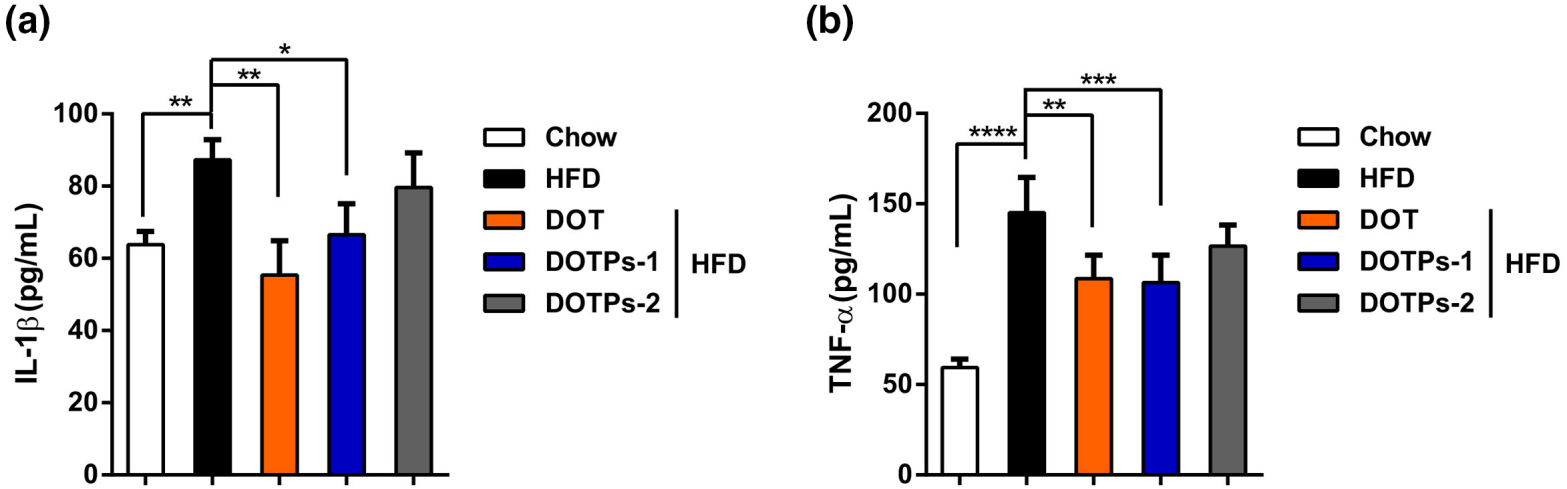
DOTPs decreased pro‐inflammatory cytokine expression levels in HFD‐fed mice. The levels of pro‐inflammatory cytokines were measured after 12 weeks of treatment as described in Figure 1. (a) The levels of serum IL‐1β and (b) serum TNF‐α were measured. Data are presented as the mean ± SD of three independent experiments (*p < .05, **p < .01, ***p < .001, ****p < .0001).

We measured the levels of the pro‐inflammatory cytokines IL‐1β and TNF‐α after 12 weeks of HFD feeding, in the absence or presence of DOT and DOTPs supplementation. The levels of IL‐1β and TNF‐α in HFD‐fed mice were significantly higher than in mice fed a chow diet (Figure 3a,b). Notably, the supplementation with DOT or DOTPs resulted in a reduction in the levels of these pro‐inflammatory cytokines in HFD‐fed mice (Figure 3a,b). These findings showed that the DOTPs inhibited inflammation in HFD‐fed mice.

### 3.4 DOTPs ameliorated HFD‐induced gut dysbiosis

**FIGURE 4:**
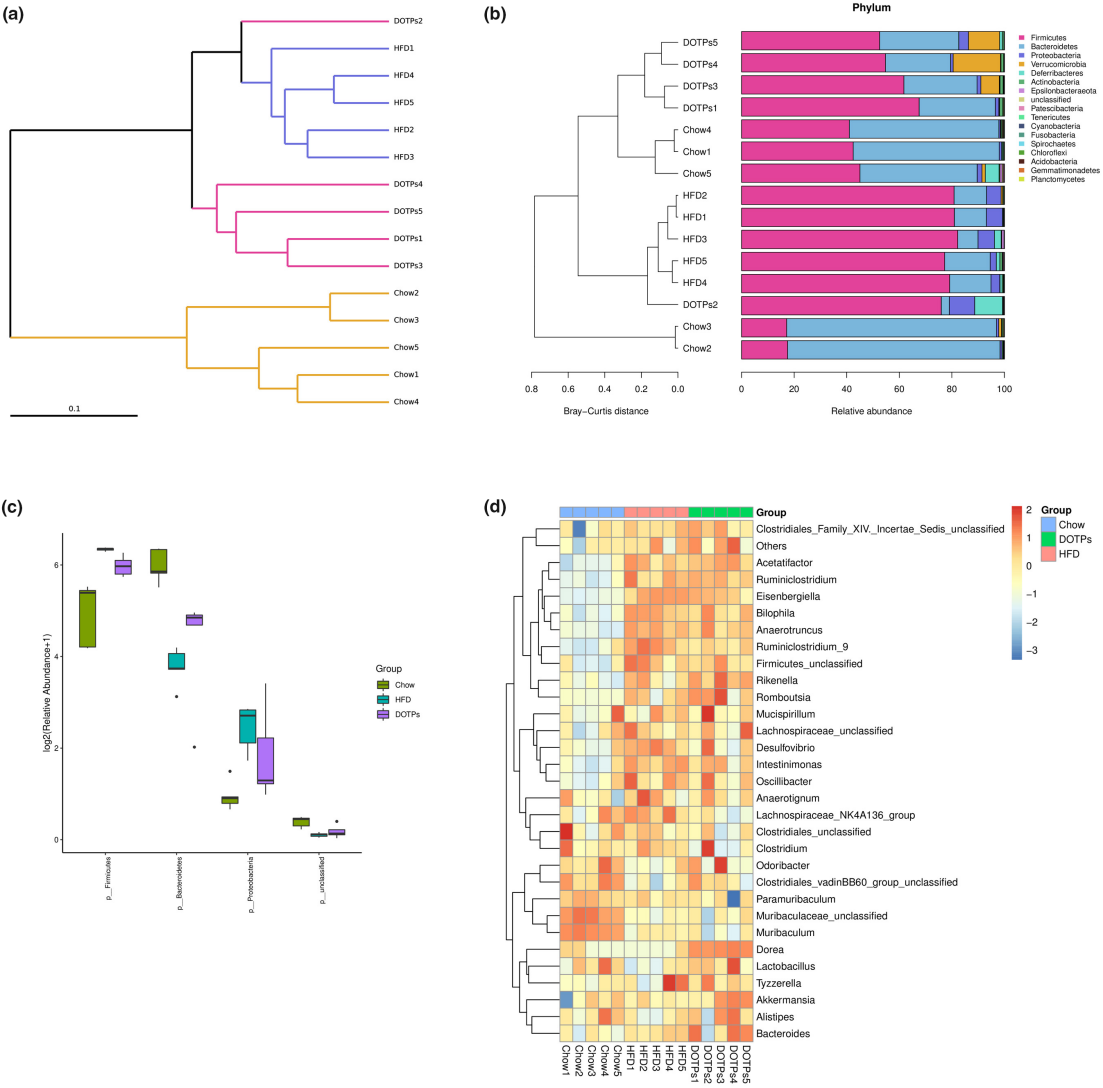
DOTPs altered the microbiota composition in HFD‐fed mice. The microbiota composition in the feces of chow‐fed and HFD mice, treated with or without 60 mg/kg DOTPs, was analyzed using next‐generation sequencing (*n* = 5 for each group). (a) The clustering analysis (UPGMA). (b) Dendrogram clustering and bacterial taxonomic profile. (c) Relative abundance of *Firmicutes*, *Bacteroidetes*, and *Proteobacteria*. (d) Heatmap of bacterial taxonomic profiling based on the top 30 dominant genera.

As previously mentioned, polysaccharides are effective prebiotics in the prevention of HFD‐induced obesity, which function by regulating gut microbiota dysbiosis. To investigate the impact of DOTPs on the gut microbiota composition, we conducted an analysis of bacterial 16S rDNA in mouse feces. As shown in Figure 4a, the clustering analysis indicated a statistically significant separation among the microbiota from the Chow, HFD, and DOTPs groups. The bacterial taxonomic profiling was conducted at the phylum level (Figure 4b). In all groups, *Firmicutes*, *Bacteroidetes*, and *Proteobacteria* were the three dominant phyla in the gut microbiota of the mice (Figure 4b). It can be concluded that the intake of an HFD changed the composition of the gut microbiota at the phylum level, according to the systematic cluster and bacterial taxonomic maps. Upon administration of DOTPs, the gut microbiota profiles in DOTPs group were separated from those of the HFD group and shared the similarity with the Chow group. Compared with the Chow group, the consumption of an HFD significantly increased the relative abundance of *Firmicutes* and *Proteobacteria* (*p* < .05) but decreased that of *Bacteroidetes* (*p* < .05) in the HFD group (Figure 4c). The administration of DOTPs could partially reverse these changes. To further assess the specific genera regulated by DOTPs, we constructed heat maps based on the top 30 dominant genera (Figure 4d). In comparison with the Chow group, the HFD group exhibited a significant increase in the relative abundance of 11 genera (*Eisenbergiella*, *Bacteroides*, *Acetatifactor*, *Ruminiclostridium_9*, *Ruminiclostridium*, *Intestinimonas*, *Romboutsia*, *Bilophila*, *Desulfovibrio*, *Rikenella*, and *Anaerotruncus*), while *Muribaculum* showed a significant decrease. In comparison with the HFD group, administration of DOTPs increased the relative abundance of two genera (*Akkermansia* and *Dorea*), while two genera (Lachnospiraceae_NK4A136_group and Ruminiclostridium_9) were significantly decreased.

### 3.5 DOTPs modulated the gut microbiota metabolism in HFD‐fed mice

**FIGURE 5:**
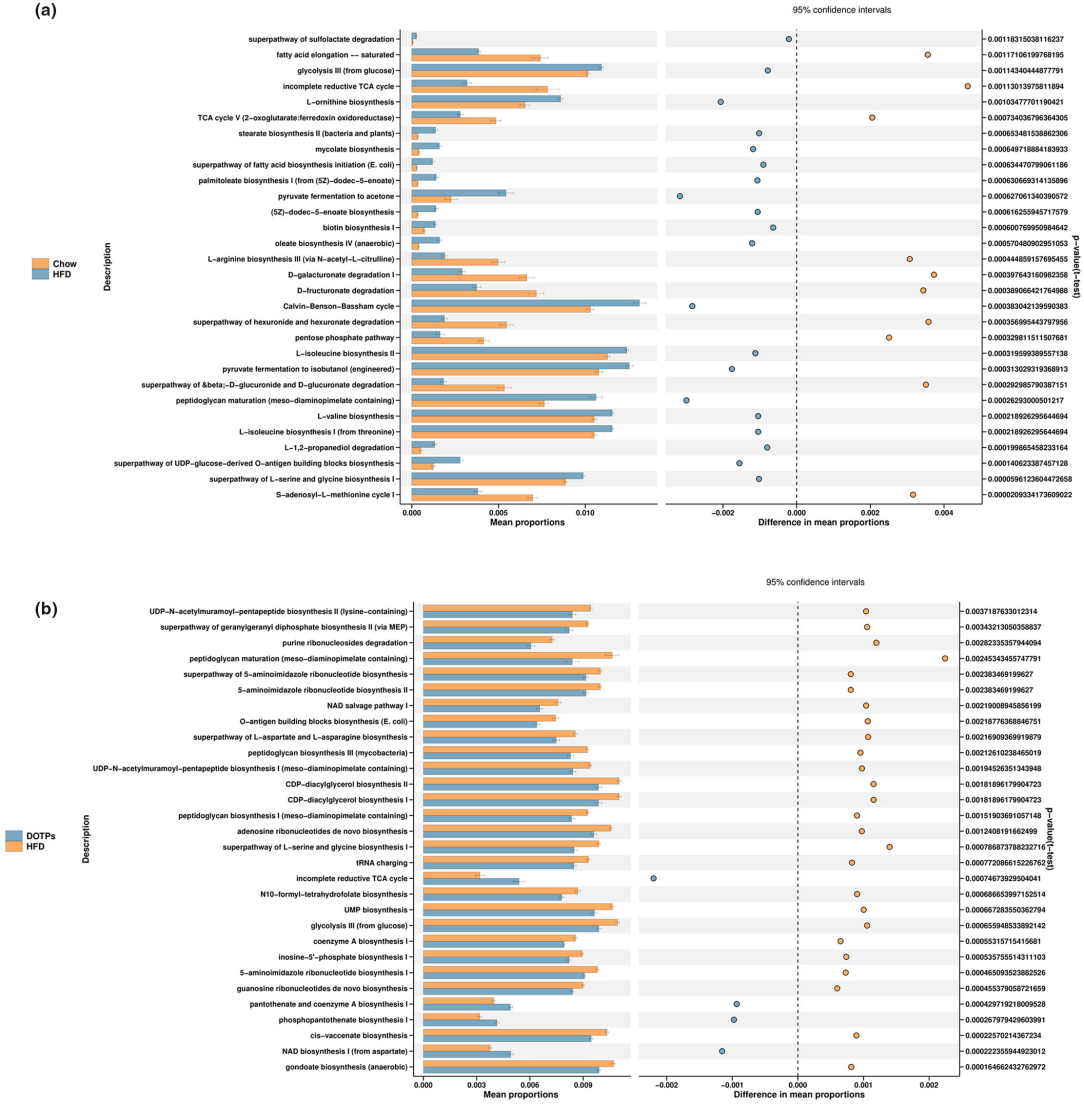
Effects of DOTPs supplementation on metabolic pathways of the gut microbiota in HFD‐fed mice. Experiments were performed as described in Figure 4. (a) Metabolic pathways of the HFD versus chow groups. (b) Metabolic pathways of the HFD versus DOTPs groups. Statistical significance was set as *p* < .05.

The phylogenetic investigation of communities by reconstruction of unobserved states (PICRUSt) method was employed to predict the metabolic pathways. To further evaluate the effect of DOTPs supplementation on the gut microbiota metabolism in HFD‐fed mice, we predicted the metabolic pathways, as shown in Figure 5. We identified 30 distinct pathways between the Chow and HFD groups (Figure 5a). Compared with the Chow group, the HFD group showed decreased activities in 10 of these pathways, such as fatty acid elongation, incomplete reductive tricarboxylic acid cycle (TCA), L‐arginine biosynthesis, D‐galacturonate degradation, D‐glucarate degradation, hexuronide and hexuronate degradation, pentose phosphate pathway, and S‐adenosyl‐L‐methionine cycle. Meanwhile, the HFD group exhibited increased levels of activity in 20 pathways, including peptidoglycan maturation, L‐serine and glycine biosynthesis, glycolysis, stearate biosynthesis, mycolate biosynthesis, pyruvate fermentation to acetone, and oleate biosynthesis. There were 30 distinct metabolic pathways identified in the HFD and DOTPs groups (Figure 5b). In comparison with the DOTPs group, the HFD group showed decreased activities in four pathways, including incomplete reductive TCA cycle, pantothenate and coenzyme A biosynthesis, phosphopantothenate biosynthesis, and nicotinamide adenine dinucleotide (NAD) biosynthesis. The other pathways, including peptidoglycan maturation, L‐serine and glycine biosynthesis, glycolysis, and uridine monophosphate (UMP) biosynthesis, were increased in the HFD group.

## 4 DISCUSSION

Some previous studies have indicated that polysaccharides extracted from TCM could decrease obesity and regulate lipid metabolism disorders in HFD mice (Chang et al., 2015; Shi et al., 2015; Wu et al., 2019). *Dioscorea opposita* Thunb. contains polysaccharides (DOTPs), which are recognized as one of its main active constituents. In the present study, we performed a series of experiments to explore the correlation between DOTPs and obesity. After 12 weeks of feeding, the body weight of mice in the HFD group was significantly higher than in the DOTPs supplementation group, without any reduction in the food intake. The administration of DOTPs also significantly decreased the weight gained, the iWAT weight, the eWAT weight, and the adipocyte size. We also measured the levels of blood glucose and insulin after 12 weeks of feeding. The insulin resistance was assessed by the HOMA‐IR index, because of the technical simplicity of this method and its close correlation with more complex tests (Ascaso et al., 2003; Beaulant et al., 2022; Kernan et al., 2016). In our study, DOTPs supplementation decreased the blood glucose levels and prevented insulin resistance in HFD‐fed mice.

Then, we explored the potential mechanisms through which DOTPs inhibited obesity in mice. Pro‐inflammatory macrophages can induce insulin resistance. Macrophages in adipose tissue have the ability to secrete a variety of inflammatory factors, including IL‐1β, TNF‐α, IL‐6, and matrix metalloproteinase‐2, which directly impair insulin action (Wellen & Hotamisligil, 2005; Wen et al., 2011). In the present study, we measured IL‐1β and TNF‐α levels in mouse serum, using an ELISA kit. In comparison with mice supplemented with DOTPs, the results indicated a significant increase in IL‐1β and TNF‐α levels in the HFD group. We found a causal relationship between the weight gained and the peripheral expression levels of inflammatory factors. Polysaccharides can regulate abnormal lipid metabolism, by reducing the expression levels of related inflammatory factors. For instance, supplementation with polysaccharides extracted from green walnut husk reduced the levels of pro‐inflammatory cytokines TNF‐α and IL‐1β, via gut microbiota regulation, which is helpful to inhibit the occurrence of obesity (Wang et al., 2021). Nevertheless, the working hypothesis is that DOTPs may also directly affect pro‐inflammatory factors to control adiposity.

The attenuation of the intestinal mucosal barrier function, induced by HFD consumption, could increase the occurrence of chronic inflammatory diseases (Schroeder et al., 2018). Previous studies have demonstrated that the gut microbiota of obese individuals or HFD‐fed mice is characterized by an increased *Firmicutes*‐to‐*Bacteroidetes* ratio, as well as elevated levels of *Proteobacteria* (Chang et al., 2015; Ley et al., 2006). Accordingly, feeding with polysaccharides from *Ophiopogon* reversed the *Firmicutes*‐to‐*Bacteroidetes* ratio and reduced obesity (Shi et al., 2015). In the present study, DOTPs reversed the HFD‐induced gut dysbiosis in obese mice. We observed that DOTPs supplementation in HFD‐fed mice restored the *Firmicutes*‐to‐*Bacteroidetes* ratio. *Bifidobacterium*, *Olsenella*, and *Lachnospiraceae_NK4A136_group* supplementation were previously reported to prevent obesity (Chen et al., 2011; Kong et al., 2019; Ma et al., 2020; Wang et al., 2020). Intriguingly, in the present study, *Bifidobacterium* and *Olsenella* were not detected, as well as *Lachnospiraceae_NK4A136_group* showed contrary results. This finding suggests that DOTPs may exert an inhibitory effect on obesity, by modulating the levels of other specific bacterial genera. *Akkermansia* has been shown to exert beneficial effects on the improvement of obesity (Cani & de Vos, 2017; Plovier et al., 2017). The protective function of *Akkermansia* on the intestinal barrier could reduce the uptake of endotoxin lipopolysaccharide into the intestinal tract and mitigate intestinal inflammation (Schneeberger et al., 2015). In contrast, *Ruminiclostridium_9* can cause abnormal lipid regulation, promoting the development of inflammation, which is related to the development of obesity (Wang, Jin, et al., 2018; Zhao et al., 2021). Our findings demonstrated that DOTPs supplementation enhanced the bacterial levels of *Akkermansia* and depleted the levels of *Ruminiclostridium_9* in HFD‐fed obese mice. The impact of DOTPs on anti‐obesity approaches could be partially due to ameliorate the gut microbiota dysbiosis.

Short‐chain fatty acids (SCFAs) represent the primary product of bacterial metabolism in the intestinal tract. SCFAs play a crucial role in regulating energy metabolism supply, improving chronic inflammatory diseases, and maintaining the dynamic balance of the intestinal environment (Sonnenburg et al., 2010). Fructooligosaccharides, a type of soluble dietary fiber, can be converted into SCFAs through their fermentation by the gut microbiota. SCFAs changed the bacterial community structure with a reduction in the abundance of *Firmicutes* and an increase in the abundance of *Bacteroidetes* (Lu et al., 2016). DOTPs supplementation in HFD‐fed mice was associated with a decreased *Firmicutes*‐to‐*Bacteroidetes* ratio. Whether DOTPs have an impact on SCFAs remains to be further studied.

It is worth noting that the glycolysis metabolic pathway was significantly downregulated under the administration of DOTPs. Previous studies have highlighted the significance of glycolysis in the development of obesity. Glycolysis upregulation, as a driver of low‐grade inflammation in obesity, culminated in IL‐1β induction (Sharma et al., 2020). In our study, the inhibition of glycolysis could be positively associated with the suppression of IL‐1β induction. Nevertheless, DOTPs could be applied as a potential prebiotic in anti‐obesity therapies.

There is still extensive research to be conducted, in order to mitigate the following limitations: (1) The polysaccharides DOTP‐B, DOTP80, and glucomannan were extracted from DOT; however, the specific polysaccharides that actually played the above‐described effects remain to be identified. (2) Activating BAT could be a promising therapy for obesity. Nevertheless, whether DOTPs exert their anti‐obesity effects through the activation of BAT remains to be clarified. (3) Our experimental results showed that DOT exerted comparable effects to DOTPs. While polysaccharides are predominantly enriched during the water‐based extraction process, we cannot exclude the possibility that other bioactive compounds present in DOT could also contribute to the observed anti‐obesity effects. Our future research will specifically address these limitations.

## 5 CONCLUSIONS

**FIGURE 6:**
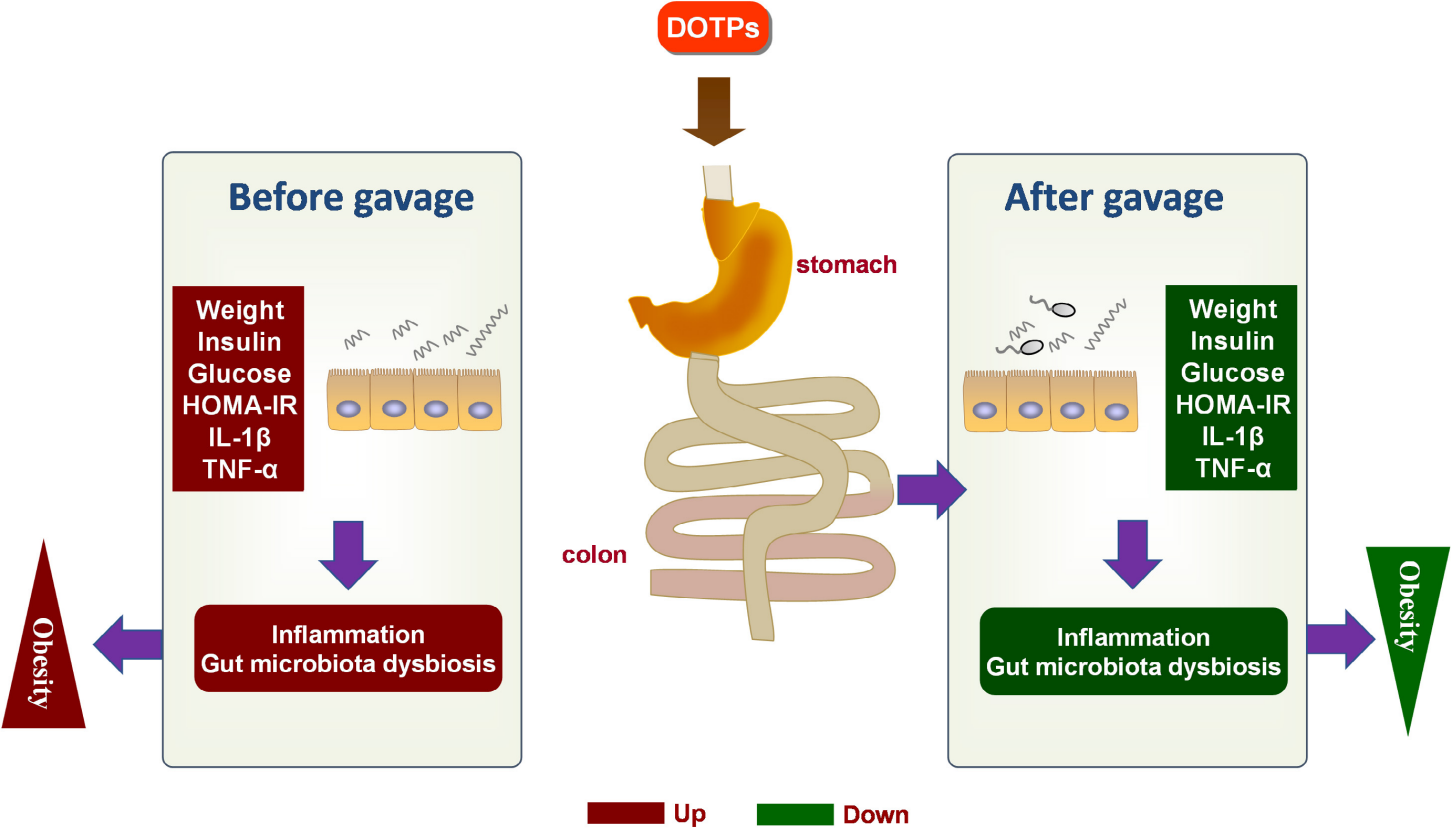
A schematic diagram summarizing the potential working model of DOTPs on anti‐obesity effects in HFD‐fed mice. The potential mechanism of DOTPs on anti‐obesity effects could provide a method of inhibiting systematic inflammation and ameliorating gut microbiota dysbiosis.

In summary, our findings indicate that DOTPs produced significant anti‐obesity effects in HFD‐fed mice (Figure 6). The modulation of the potential underlying mechanisms could be a method of inhibiting systematic inflammation and ameliorating gut microbiota dysbiosis. DOTPs supplementation could enrich specific beneficial microbial genera, inhibit specific harmful microbial genera, and reduce disadvantageous metabolic pathways related to the development of obesity. Collectively, our findings suggest that DOTPs may be used as a potential prebiotic in preventing diet‐induced obesity.

## AUTHOR CONTRIBUTIONS

**Sheng‐Nan Li:** Conceptualization (lead); formal analysis (lead); funding acquisition (lead); investigation (lead); writing – original draft (lead). **Dian‐Long Zhang:** Data curation (supporting); investigation (equal). **Zhen‐Hui Wang:** Conceptualization (supporting); supervision (equal). **Wen‐Ting Song:** Funding acquisition (supporting); methodology (equal). **Wen‐Bo Chen:** Funding acquisition (supporting); writing – review and editing (equal). **Ge‐Li Hu:** Methodology (supporting). **Lu‐Ying Han:** Methodology (supporting). **Jing‐Chun Zhou:** Project administration (supporting).

## CONFLICT OF INTEREST STATEMENT

The authors declare that they do not have any conflict of interest.

## ETHICS STATEMENT

This study was approved by the Medical Ethics Committee of Henan Polytechnic University (approval code: HPU‐MEC‐2020‐04). All procedures used in this study complied with the Guide for the Care and Use of Laboratory Animals of Henan Polytechnic University.

## Data Availability

The data that support the findings of this study are available from the corresponding author upon reasonable request.
